# Molecular World Today and Tomorrow: Recent Trends in Biological Sciences 2.0

**DOI:** 10.3390/ijms25053070

**Published:** 2024-03-06

**Authors:** Wajid Zaman

**Affiliations:** Department of Life Sciences, Yeungnam University, Gyeongsan 38541, Republic of Korea; wajidzaman@yu.ac.kr

Molecular techniques have become influential instruments in biological study, transforming our comprehension of life at the cellular and genetic levels [1]. The Special Issue "Molecular World Today and Tomorrow: Recent Trends in Biological Sciences 2.0" examines current trends in biological sciences and the impact of advanced techniques on scientific advancements. The problem highlights how molecular techniques can greatly aid in the advancement of focused medication creation and disease diagnostics. Combining molecular phylogenetic and omics approaches with expression and pathway analysis, we seek to improve our comprehension of molecular mechanisms and stress responses in biological systems [2]. This collaboration represents significant progress in genomics, proteomics, and phylogenetic methods, providing new opportunities for study and innovation.

Advancements in omics technologies, including transcriptomics, proteomics, and genomics, have significantly improved our capacity to recognize and describe biological entities [2,3]. Researchers currently use systematic methods to uncover genetic variations, forecast ecological habitats, and clarify taxonomic intricacies [4,5]. Omics data offer a thorough perspective on the molecular world, ranging from analyzing microbial populations to uncovering the genetic foundations of diseases. Integrating molecular phylogenetics with omics techniques enhances our comprehension of evolutionary connections. Researchers investigate molecular pathways across many taxonomic groups by integrating sequence data with expression profiles [6,7]. This synergy influences both evolutionary history and functional adaptations, as well as stress responses. 

Molecular data drives precision medicine [8,9]. Genomics and proteomics play a crucial role in guiding individualized treatments by identifying therapeutic targets and predicting medication responses [10,11]. Furthermore, diagnostic tests utilizing molecular markers improve the diagnosis and prediction of diseases. As we decode the complexities of individual genomes, the future shows potential for personalized interventions. The study in this Special Issue highlights the significance of interdisciplinary approaches in addressing intricate biological issues through collaboration. It unites specialists from several disciplines, such as molecular biology, genetics, biochemistry, biophysics, and computational biology, to promote a comprehensive comprehension of life at the molecular scale. This collaborative effort is crucial for the integration of knowledge and the acceleration of scientific discoveries (Figure 1).

The edition features 20 papers, including 3 comprehensive review articles, a brief report, 1 communication, and 15 research articles. The articles in the Special Issue emphasize the importance of molecular data in promoting targeted medicines, highlighting the vital significance of molecular sciences in medicine. This interdisciplinary method highlights the significant impact of molecular approaches in research and clinical environments, presenting new opportunities for customized medicine and treatments.

In conclusions, this Special Issue demonstrates the dynamic and interdisciplinary nature of molecular sciences. It motivates researchers to expand the current knowledge and investigate new areas, assuring the ongoing development of the biological sciences field and its contribution to our understanding of life’s molecular foundation. The compilation showcases the present status of molecular sciences and paves the way for future progress, encouraging further inquiry and invention in this captivating discipline.

## Figures and Tables

**Figure 1:**
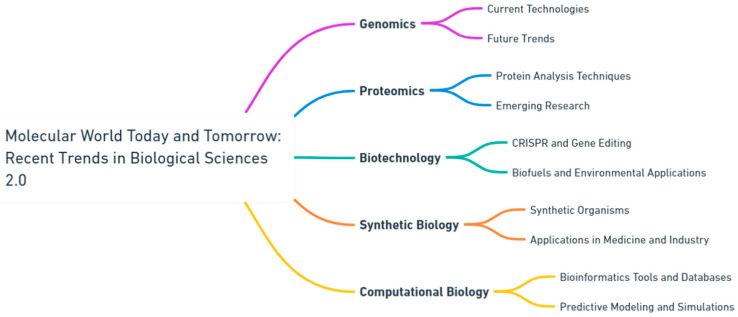
Navigating the frontier: current and future perspectives in biological sciences (generated by OpenAI's Whimsical Diagrams GPT).
